# Supplementary material for: Comparative analysis of genetic diversity and differentiation of cauliflower (Brassica oleracea var. botrytis) accessions from two ex situ genebanks
Source: PLoS One. 2018 Feb 8;13(2):e0192062. doi: 10.1371/journal.pone.0192062 (PMC5805252; doi:10.1371/journal.pone.0192062)
Supplement: S1 File — (PDF) [file pone.0192062.s001.pdf]

Supplementary Material
Comparative analysis of genetic diversity and differentiation of cauliflower (*Brassica oleracea* var. *botrytis*) accessions from two *ex situ* genebanks

Yousef et al.

January 22, 2018

1 Supplementary Figures

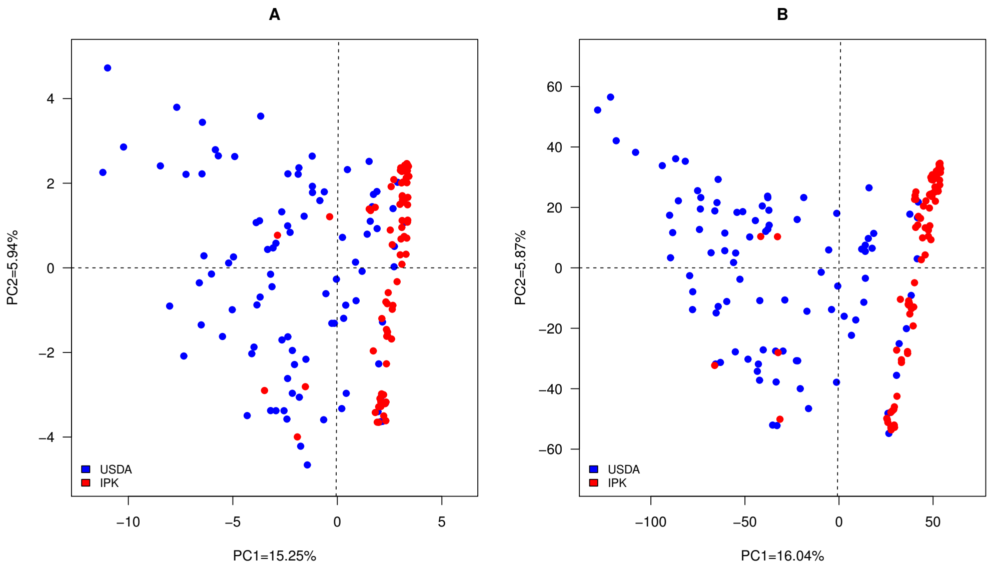

Figure A: Comparison of the principal component analysis (PCA) of 174 cauliflower accessions. (A) PCA based on the dataset with missing data and (B) using genotypes imputed with fastPHASE.

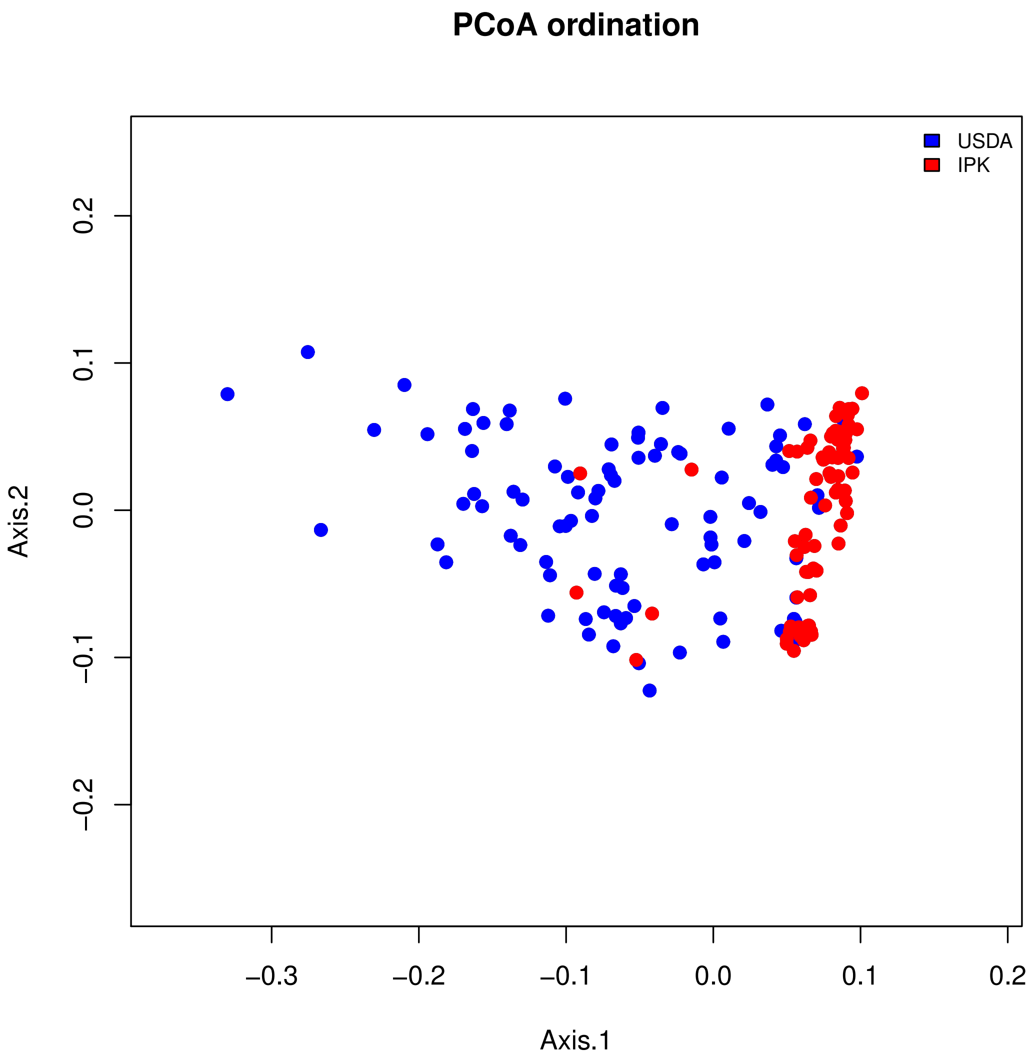

Figure B: PCoA of pairwise F_{st} values between individuals.

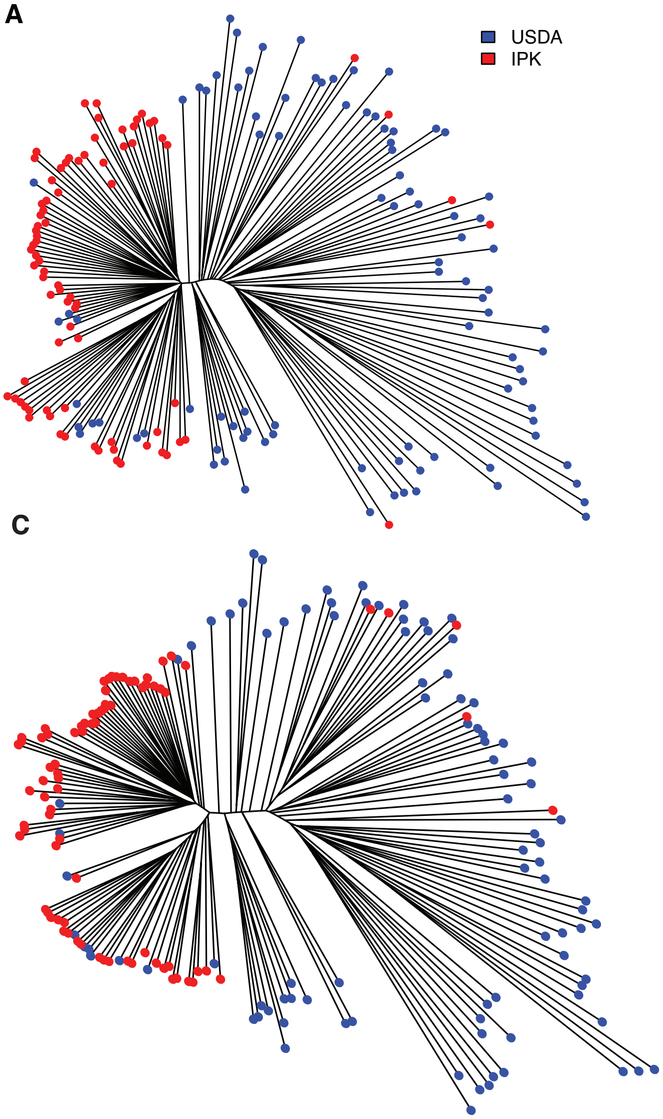

Figure C: Neighbor-joining tree of 174 accessions. The tree is based on the pairwise distance matrix. (A) and (B) Analysis data with missing values. (C) and (D) analysis with imputed data. (A) and (C) Accessions are represented by different colors according to the genebank or origin. (B) and (D) Accessions are represented according to the country of origin.

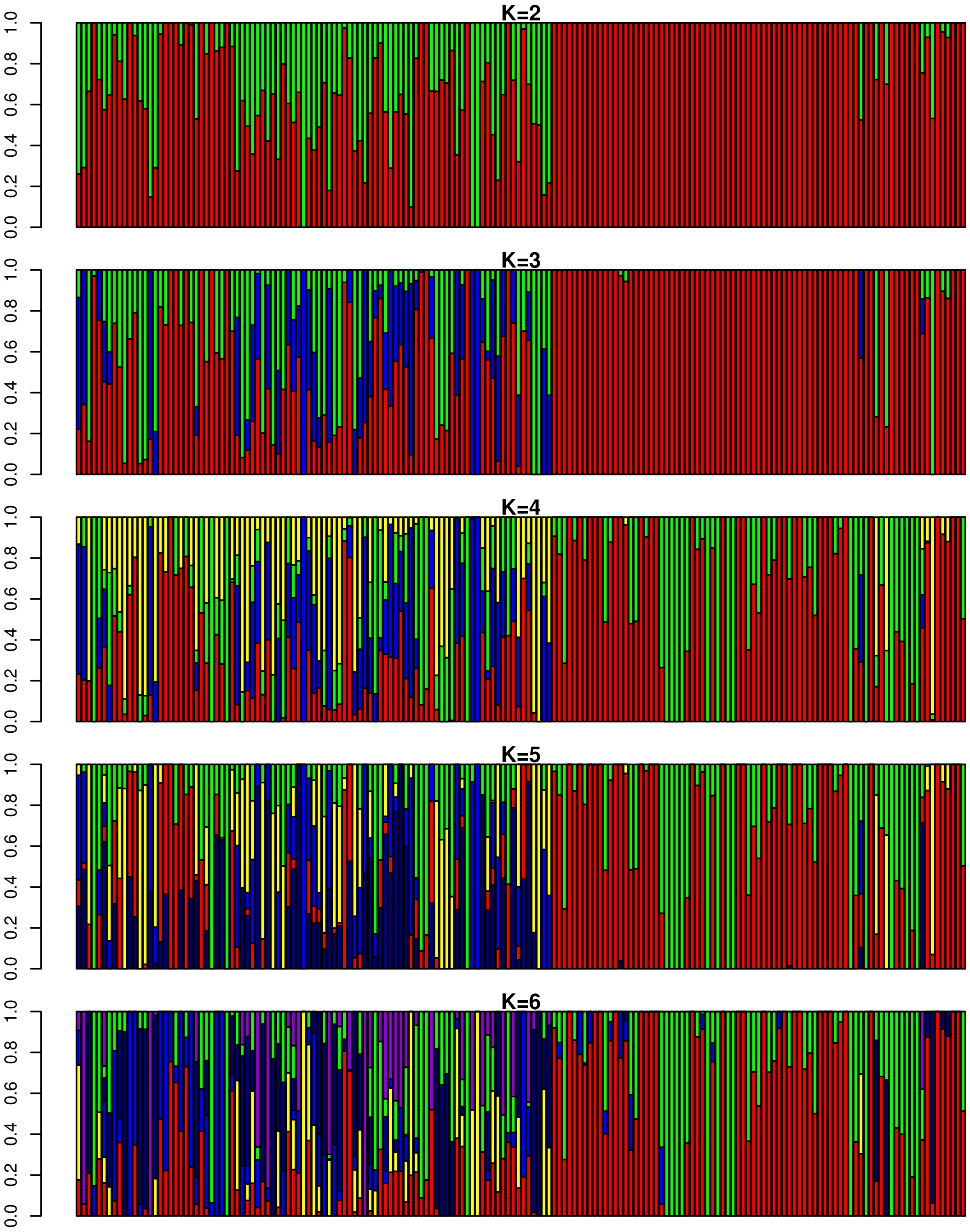

Figure D: Population structure analysis of 174 cauliflower accessions with ADMIXTURE based on SNPs with missing data.

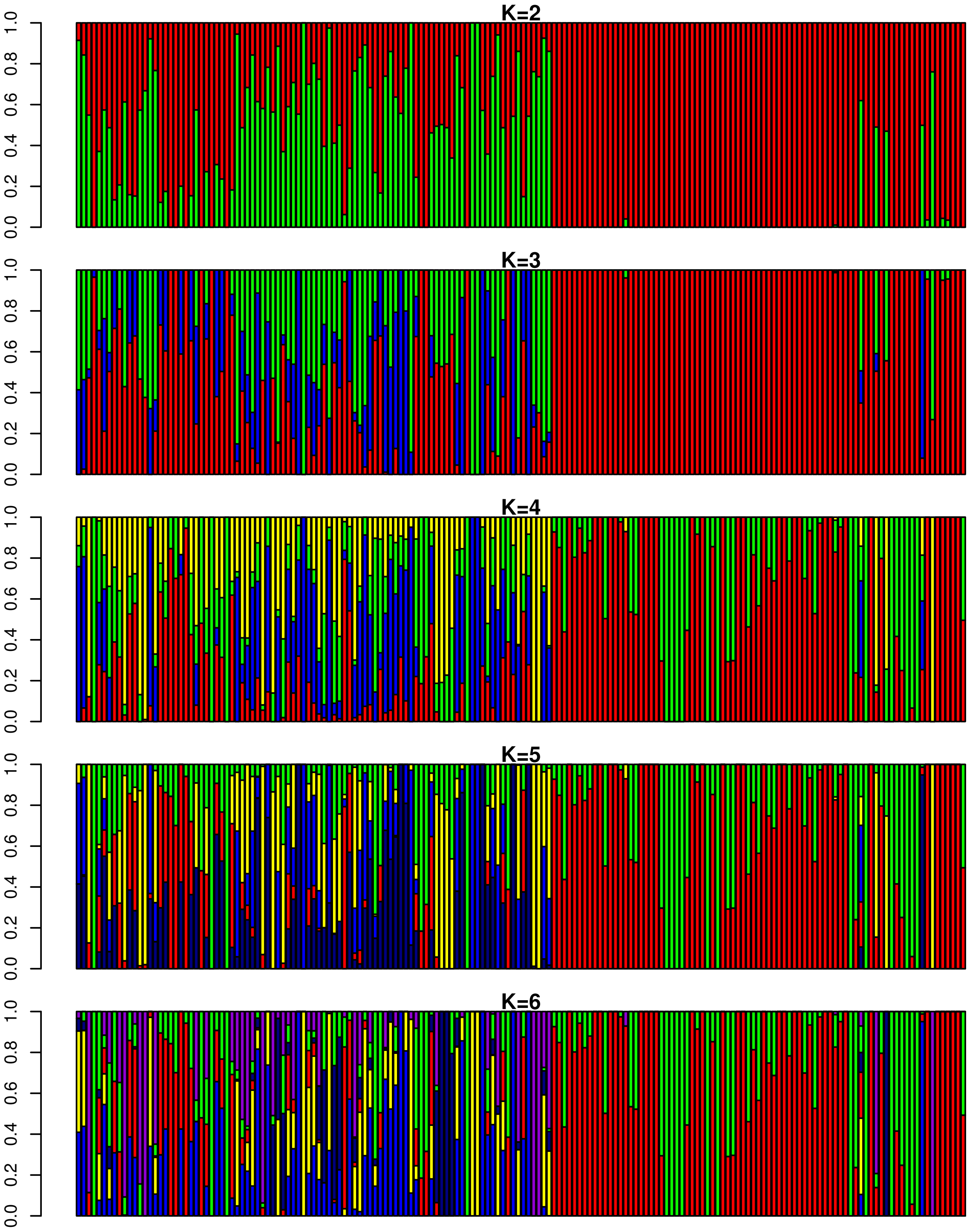

Figure E: Population structure analysis of 174 cauliflower accessions with ADMIXTURE based on SNPs with imputed data.

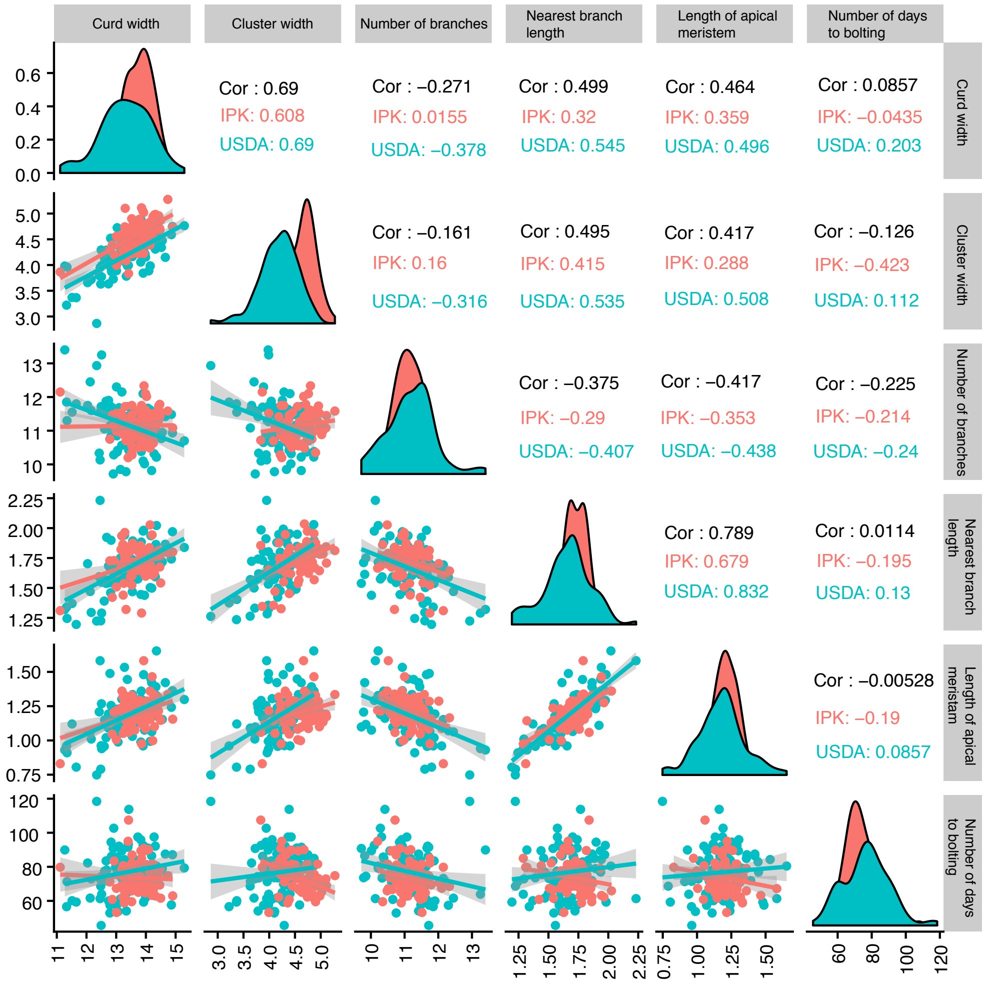

Figure F: Correlation analysis of six phenotypic traits of cauliflower. The lower triangle shows scatterplots of pairs of traits, the central axis the distribution of trait vaules and the upper triangle the correlation coefficients.

2 Supplementary Note

Calculation of the distance matrix

After SNP calling, we know which SNP allele each genotype has at each position. For the calculation of a simple genetic distance matrix, we counted the pairwise differences between each pair of genotype and each position. We will explain the procedure with an example:

Consider two genotypes and their SNP sequence at five positions (pos):

	pos1	pos2	pos3	pos4	pos5
G1:	A/A	T/G	N/N	G/C	C/C
G2:	A/A	T/T	G/G	G/C	G/G

At the first position (pos1) both genotypes are homozygous with A/A. At the second position G1 is heterozygous with T and G while G2 is homozygous with T/T. At the third position there was no information for G1 (N/N). At the fourth position, both genotypes are heterozygous and at the fifth position, both are homozygous, but for different nucleotides.

To calculate the distance, we look at each position and count the number of pairwise differences:

$$d(\text{pos1}) = 4 \times d(\text{A,A}) \qquad = 4 \times 0 = 0$$
$$d(\text{pos2}) = 2 \times d(\text{T,T}) + 2 \times d(\text{G,T}) \qquad = 2 \times 0 + 2 \times 1 = 2$$
$$d(\text{pos3}) = 0$$

We do not consider $d(pos)3)$ because there is a 'N' in one of the genotypes (pairwise exclusion):

$$d(\text{pos4}) = d(\text{G,G}) + d(\text{G,C}) + d(\text{C,C}) \qquad = 0 + 1 + 0 + 1 = 2$$
$$d(\text{pos5}) = 4 \times d(\text{C,G}) \qquad = 4 \times 1 = 4$$

The total distance is then

$$\frac{d(\text{G1,G2})}{2} = \sum_{i=1}^{5} d(\text{pos}_i) = \frac{0+2+0+2+5}{2} = 4$$

We took half of the distance because we calculated the distance at the position as if there were two positions, which is not the case because we consider SNPs.
